# Emergency risk stratification using the TyG index: a multi-center cohort study on nonlinear association with 28-day mortality among critically ill patients transferred from the ED to the ICU

**DOI:** 10.3389/fmed.2025.1605843

**Published:** 2025-07-30

**Authors:** Zhenhua Huang, Jianshe Bu, Ke Yu, Wanjie Gu, Haiyan Yin

**Affiliations:** ^1^Department of Emergency Medicine, The First Affiliated Hospital of Jinan University, Guangzhou, China; ^2^Department of Emergency Medicine, The First Affiliated Hospital of Shenzhen University, Shenzhen Second People’s Hospital, Shenzhen, China; ^3^Department of Gynaecology, Shenzhen People’s Hospital (The Second Clinical Medical College, Jinan University; The First Affiliated Hospital, Southern University of Science and Technology), Shenzhen, China; ^4^Department of Pulmonary and Critical Care Medicine, The First Affiliated Hospital of Shenzhen University, Shenzhen Second People’s Hospital, Shenzhen, China; ^5^Department of Intensive Care Unit, The First Affiliated Hospital of Jinan University, Guangzhou, China

**Keywords:** triglyceride-glucose index, emergency critical care, risk stratification, insulin resistance, mortality prediction, ICU admission

## Abstract

**Background:**

In the emergency department (ED), rapid risk stratification of critically ill patients is essential for timely intervention. The triglyceride-glucose (TyG) index, a simple marker of insulin resistance, may aid in early mortality prediction, but its utility in ED-to-ICU patients remains unexplored.

**Methods:**

Using data from the eICU Collaborative Research Database, we conducted a retrospective multicenter cohort study of 11,593 ED-to-ICU critically ill patients. The TyG index was calculated at ED presentation. The primary outcome was 28-day all-cause mortality. Multivariable Cox regression, restricted cubic splines, and sensitivity analyses were performed to assess associations.

**Results:**

Among patients (mean age 63.6 ± 15.7 years, 57.3% male), 28-day mortality was 6.96%. The relationship between the TyG index and mortality was nonlinear, featuring a critical threshold at a TyG index value of 9.84. Below this cutoff, each unit increase in TyG index significantly elevated mortality risk (HR 1.47, 95% CI 1.20–1.69, *p* < 0.0001), while above it, the risk plateaued (HR 1.04, 95% CI 1.03–1.05, *p* = 0.097). The association remained robust after adjustment for confounders (adjusted HR 1.19, 95% CI 1.04–1.35, *p* = 0.0089) and across sensitivity analyses.

**Conclusion:**

The TyG index, readily obtainable at ED presentation, provides emergency clinicians with a practical tool for early mortality risk stratification in critically ill patients. Its nonlinear association with 28-day mortality suggests a saturation effect, enabling rapid identification of high-risk patients who may benefit from intensified monitoring and intervention.

## Introduction

The majority of critically ill patients admitted to the intensive care unit (ICU) are transferred from the emergency department (ED) (1). Recent statistics indicate that the proportion of patients transferred from the ED to the ICU has increased from 0.9 to 1.6% of all ED visits, a trend further exacerbated by the growing aging population (2). Some cohorts indicate that critically ill ED patients (e.g., sepsis, polytrauma, acute respiratory failure) face high mortality risks, with pre-ICU mortality reaching 34% (3). Another dataset shows that from 2014 to 2015, a total of 200,859 admissions of critically ill patients were recorded in 208 hospitals in the United States. Of these, 41.24% were from the emergency department, with an in-hospital mortality rate of 8.96% (4). This data highlights that in the emergency department, early identification and timely intervention in the first hour are essential for a better outcome.

Traditional ICU scoring systems (APACHE II, SOFA) lack practicality in the ED setting. These tools require complex parameters often unavailable during initial ED evaluation, delaying risk assessments (5). For example, delays in ICU transfer increase mortality by 27% in septic shock patients (6). Additionally, these scores fail to account for metabolic dysregulation—a key driver of critical illness progression (7). The ED demands simple, rapid biomarkers that integrate pathophysiological insights to enable real-time decisions.

Insulin resistance (IR), a hallmark of metabolic crisis, is prevalent in critically ill ED patients and correlates with multiorgan failure (8–12). The triglyceride-glucose (TyG) index—calculated using readily available ED labs (fasting triglycerides and glucose)—provides a pragmatic IR measure (13–15). Emerging evidence links elevated TyG index to adverse outcomes in acute myocardial infarction, cardiometabolic medicine, major adverse cardiovascular events, stroke and sepsis (16–20), suggesting its utility for ED risk stratification. Notably, a TyG index >11.18 predicts 48% higher mortality in acute kidney injury patients (21), aligning with the ED’s need for actionable thresholds.

However, existing studies are limited by small ED-specific cohorts and insufficient adjustment for resuscitation confounders (21–24). To address this gap, we analyzed 11,593 ED-to-ICU patients from the multicenter eICU-CRD. We aimed to: (1) Validate the TyG index’s prognostic value for 28-day mortality in ED- to-ICU patients; (2) Identify ED-actionable risk thresholds to optimize early interventions.

## Methods

### Study design

This retrospective multicenter cohort study analyzed ED-to-ICU patients using the eICU-CRD (2014–2015), comprising 208 U.S. hospitals (4, 25). The database’s automated data capture from ED admission through ICU care ensured fidelity to real-world emergency workflows. In terms of data storage and privacy, all data were automatically stored through Philips Healthcare’s eICU program, with anonymization processes in place to ensure compliance with the Health Insurance Portability and Accountability Act (HIPAA). Data access was granted after completing the Collaborative Institutional Training Initiative (CITI) program’s “Research with Data or Samples Only” course, thus exempting the study from review by the Massachusetts Institute of Technology Institutional Review Board (Record ID: 49995491), and waiving the requirement for informed consent. This study strictly adhered to the ethical principles outlined in the Declaration of Helsinki.

### Participants

**Figure 1:**
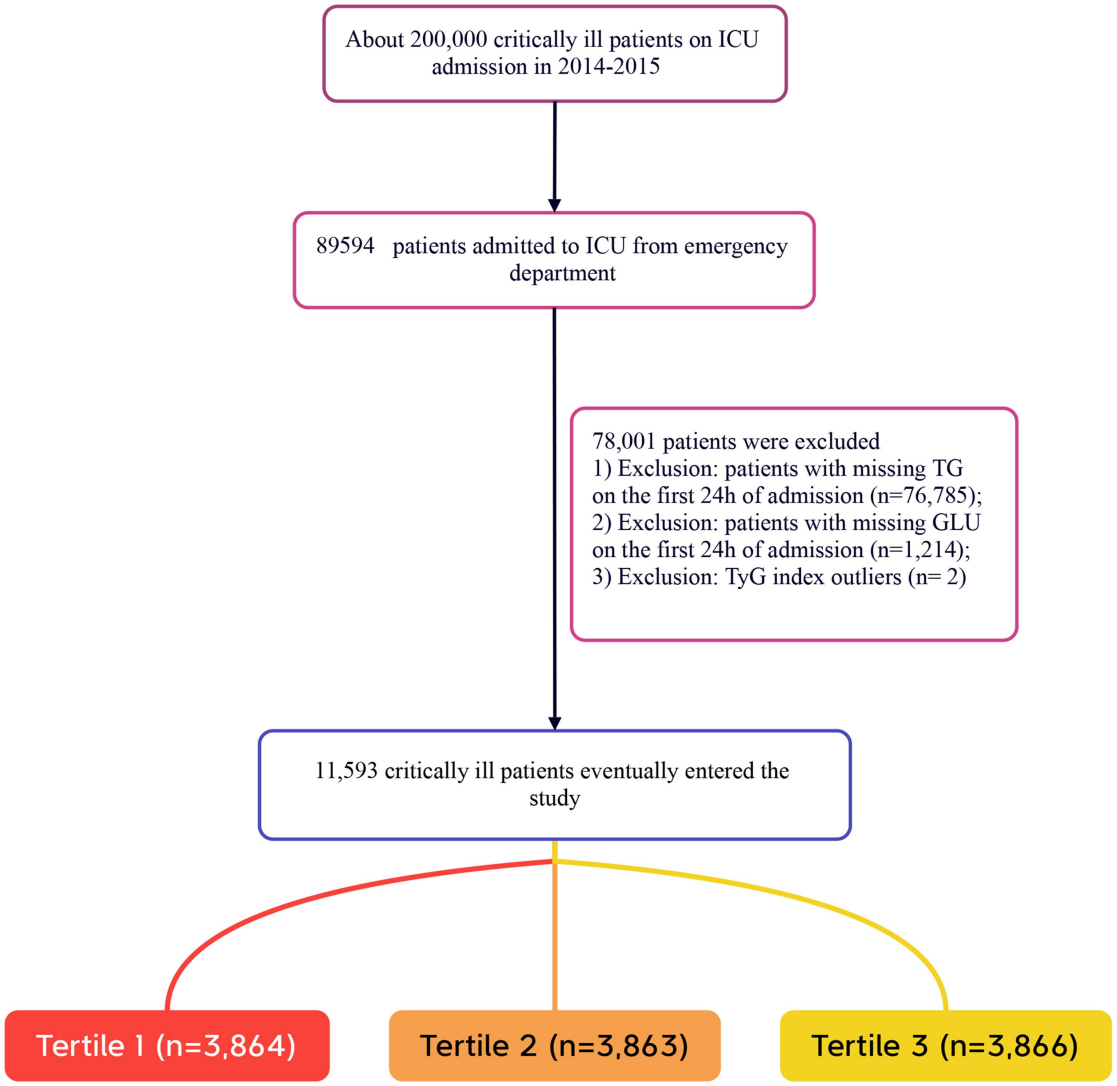
Flow chart of study population. ICU, intensive care unit.

Patients were included in the study if they met the following criteria: (1) adults aged 18 years and older; (2) individuals who were critically ill and transferred from the ED to the ICU. The exclusion criteria were as follows: (1) patients lacking triglyceride (TG) data on the day of admission; (2) patients lacking fasting plasma glucose (FPG) data on the day of admission; (3) patients with extreme TyG index values (defined as values beyond three standard deviations from the mean) (26, 27). In this study, the term “Caucasian” refers to individuals of European descent who are non-Hispanic white. A total of 11,593 patients were included in the study, and they were categorized into three groups based on the tertiles of the TyG index measured on the day of admission. The detailed patient selection process is illustrated in Figure 1.

### Variables and outcomes

ED admission variables (first 24 h): Demographics, vital signs, lab values (total cholesterol [TC], TG, high-density lipoprotein cholesterol [HDL], low-density lipoprotein cholesterol [LDL], etc.), and comorbidities (ICD-9 codes: diabetes mellitus [DM], chronic obstructive pulmonary disease [COPD], chronic heart failure [CHF] and acute myocardial infarction [AMI]). ED-relevant severity scores (SOFA, GCS, APACHE IV) were extracted to benchmark against TyG performance. The TyG index was calculated based on initial FPG and TG levels, using the formula: ln [TG (mg/dL) × FPG (mg/dL)/2] (28–30). The TyG index was computed using initial ED labs to simulate real-time risk assessment. Primary outcome: 28-day all-cause mortality post-ED admission—a critical endpoint for evaluating ED risk stratification accuracy.

### Statistical analysis

Continuous variables that followed a normal distribution were expressed as mean ± standard deviation (SD), while non-normally distributed continuous variables were described using median and interquartile range (IQR). Categorical data were presented as counts and percentages. Continuous variables were compared using the student’s t-test based on the tertiles of the TyG index, while categorical variables were analyzed using Pearson’s chi-square test or Fisher’s exact test.

We employed Cox proportional hazards models to estimate the association between the TyG index and 28-day mortality, presenting results as hazard ratios (HR) with 95% confidence intervals (CI) to illustrate regression estimates and adjusted results. Confounding factors were selected based on their association with the outcome of interest or if their effect estimates changed by more than 10%, in addition to being chosen based on clinical judgment. Consequently, we adjusted for the following covariates: gender, age, ethnicity, BMI, SOFA score, GCS score, APACH IV, BUN, Scr, TC, HDL, COPD, CHF, AMI, and DM.

Additionally, Cox regression models with cubic splines were utilized to delve into the nonlinear relationships between the TyG index and all-cause mortality. We subsequently employed a piecewise linear regression model to examine the threshold effect of the TyG index on mortality, and we conducted likelihood ratio tests to compare the single-segment linear regression model with the piecewise linear model.

To assess the robustness of our results, we performed sensitivity analyses, subgroup analyses, and interaction analyses. We calculated E-values to explore the potential impact of unmeasured confounding factors on the association between the TyG index and 28-day all-cause mortality. The E-value quantifies the magnitude of an unmeasured confounder needed to fully account for the observed association between the TyG index and 28-day all-cause mortality (31). A two-tailed alpha level was set at 0.05. All statistical analyses were conducted using EmpowerStats (X&Y Solutions, Inc., Boston, MA, USA)^1^ and R software version 3.6.1.^2^

## Results

### Baseline characteristics of the included participants

**Figure 2:**
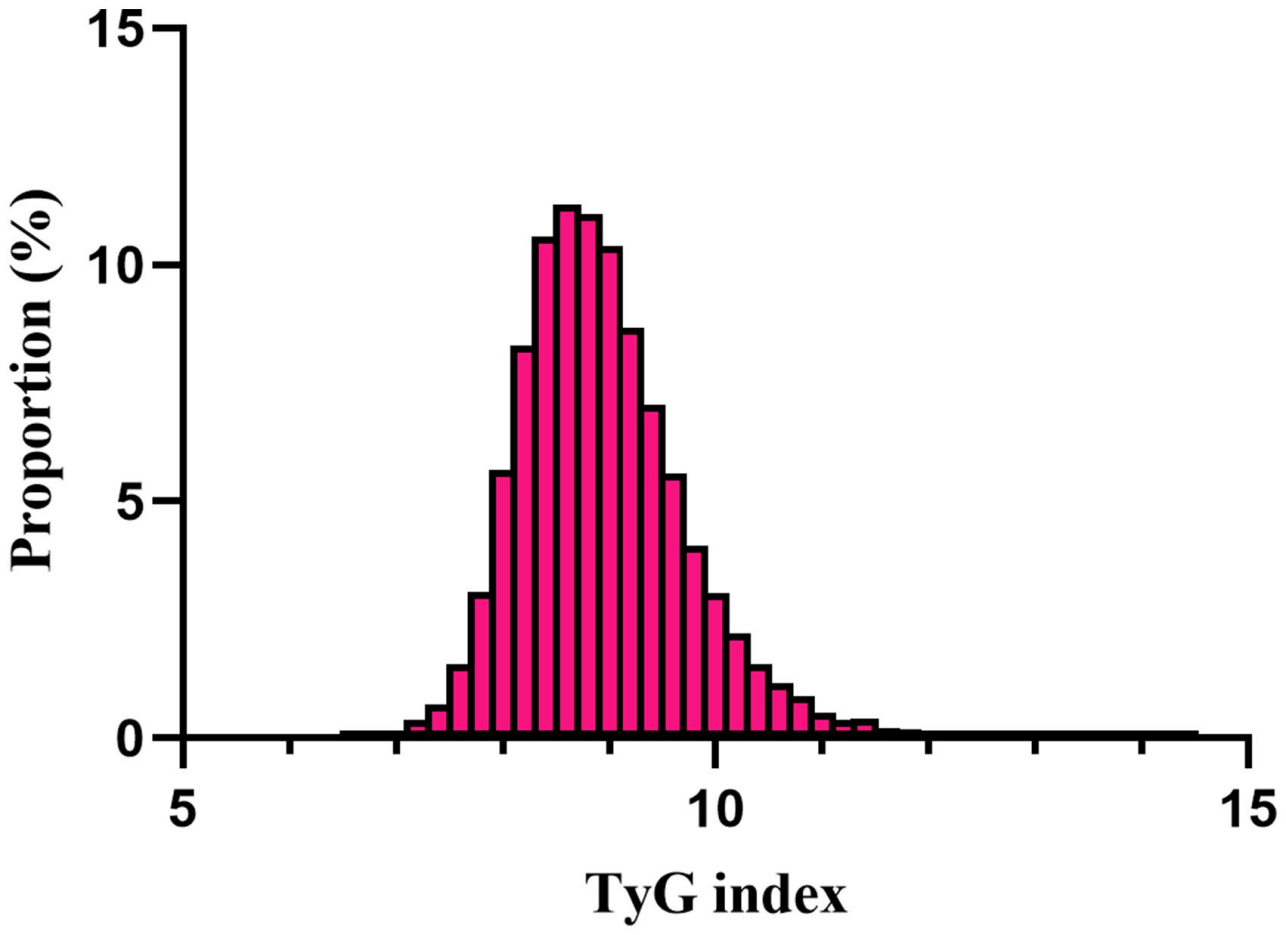
Distribution of TyG index. It presented a normal distribution, ranging from 6.54 to 11.39, with a mean of 8.96.

A total of 11,593 patients met the inclusion criteria in the eICU database (see Figure 1). The average age of the patients was 63.61 ± 15.65 years, with 6,646 males, accounting for approximately 57.33% of the cohort. The baseline TyG index values ranged from 6.54 to 11.39, with a mean of 8.96 (see Figure 2).

**Figure 3:**
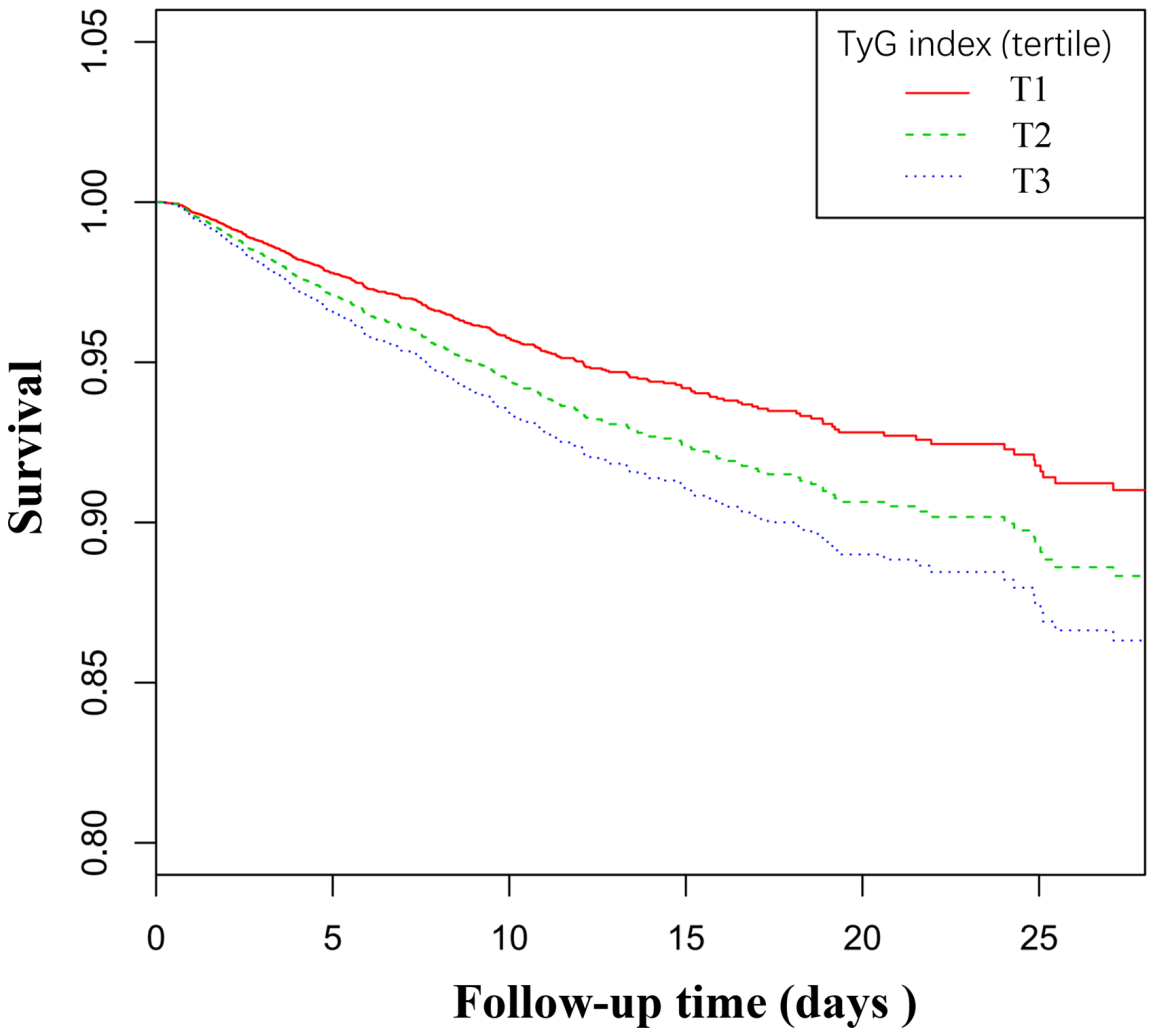
Kaplan–Meier curves for all-cause mortality. The probability of all-cause mortality increased progressively with a rising TyG index.

Table 1 presents the baseline characteristics of the patients grouped by the tertiles of the TyG index. As indicated in Table 1, there is a discernible trend in baseline characteristics and clinical outcomes with increasing tertiles of the TyG index. Higher levels of the TyG index were associated with younger age, increased BMI, a higher proportion of males, and an increased percentage of Caucasians. In terms of biochemical parameters, levels of FPG, BNU, and Scr showed an upward trend with an increasing TyG index, while HDL levels decreased. The TG and TC levels were significantly elevated in the third tertile. Clinically concerning trends were observed, as the incidence of complications such as DM, COPD, CHF, AMI, and mortality rates increased with higher TyG index tertiles. Additionally, the hospital lengths of stay tended to be longer. The 28-day all-cause mortality rate also increased with rising TyG index tertiles. Figure 3 displays the Kaplan–Meier curves for 28-day all-cause mortality. The probability of mortality progressively increased with a rising TyG index.

**Table 1:** The baseline characteristics of participants according to tertiles of TyG index.

Characteristics	T1 (6.54–8.57)	T2 (8.57–9.16)	T3 (9.16–11.39)	*p*-value
Age (year)	66.52 ± 16.60	64.86 ± 14.85	59.46 ± 14.55	<0.001
BMI (kg/m^2^)	27.60 ± 7.51	29.39 ± 7.85	31.38 ± 8.22	<0.001
Gender				0.207
Female, *n* (%)	1,693 (43.81%)	1,625 (42.08%)	1,627 (42.10%)	
Male, *n* (%)	2,171 (56.19%)	2,237 (57.92%)	2,238 (57.90%)	
Ethnicity				<0.001
Caucasian, *n* (%)	2,714 (70.88%)	2,821 (73.75%)	2,752 (72.17%)	
African-American, *n* (%)	633 (16.53%)	508 (13.28%)	476 (12.48%)	
Hispanic, *n* (%)	226 (5.90%)	243 (6.35%)	304 (7.97%)	
Asian, *n* (%)	166 (4.34%)	166 (4.34%)	182 (4.77%)	
Native American, *n* (%)	21 (0.55%)	17 (0.44%)	29 (0.76%)	
Unknown, *n* (%)	69 (1.80%)	70 (1.83%)	70 (1.84%)	
SOFA score, median (IQR)	2.0 (1.00–3.0)	1.0 (0.0–3.0)	1.00 (0.0–4.0)	<0.001
GCS score, median (IQR)	15.0 (13.0–15.0)	15.0 (13.0–15.0)	15.0 (12.0–15.0)	<0.001
APACHE IV score, median (IQR)	47.0 (35.0–62.0)	46.0 (34.0–62.0)	48.0 (34.0–66.0)	<0.001
BUN, median (IQR), (mmol/L)	16.0 (12.0–24.0)	17.0 (12.0–24.0)	18.0 (13.0–29.0)	<0.001
Scr, median (IQR), (mg/dL)	0.90 (0.71–1.23)	0.95 (0.76–1.27)	1.00 (0.79–1.47)	<0.001
FPG, median (IQR), (mg/dL)	104.0 (92.0–123.0)	121.0 (104.0–148.0)	173.0 (127.0–247.0)	<0.001
TC, (mg/dL)	140.65 ± 41.14	155.15 ± 44.42	176.00 ± 64.00	<0.001
TG, median (IQR), (mg/dL)	67.0 (54.0–83.0)	112.0 (92.0–136.0)	194.0 (144.0–268.0)	<0.001
HDL, (mg/dL)	47.99 ± 17.65	41.14 ± 14.83	35.99 ± 13.26	<0.001
Medical history				
DM, *n* (%)	270 (6.99%)	339 (8.78%)	682 (17.64%)	<0.001
COPD, *n* (%)	217 (5.62%)	210 (5.44%)	172 (4.45%)	0.044
CHF, *n* (%)	373 (9.65%)	336 (8.70%)	292 (7.55%)	0.004
AMI, *n* (%)	646 (16.72%)	883 (22.86%)	885 (22.89%)	<0.001
28-day hospital mortality				<0.001
No, *n* (%)	3,654 (94.57%)	3,603 (93.27%)	3,529 (91.28%)	
Yes, *n* (%)	210 (5.43%)	260 (6.73%)	337 (8.72%)	
Hospital LOS, median (IQR), d	4.00 (2.40–7.09)	3.87 (2.20–7.27)	3.83 (2.14–7.34)	0.016

Continuous variables are summarized as mean (SD) or median (trisection interval); categorical variables are presented as percentages (%). BMI, body mass index; COPD, chronic obstructive pulmonary disease; AMI, acute myocardial infarction; ICU, intensive care unit; LOS, length of stay; APACHE IV, acute physiology and chronic health evaluation IV; BUN, blood urea nitrogen; Scr, serum creatinine; SOFA, sequential organ failure assessment; GCS, Glasgow coma scale; FPG, fasting plasma glucose; TC, total cholesterol; TG, triglycerides; HDL, high-density lipoprotein; CHF, chronic heart failure; DM, diabetes mellitus.

### Unadjusted association between baseline variables and 28-day all-cause mortality

The univariate analysis indicated positive associations between the TyG index and 28-day all-cause mortality. In addition, we also indicated positive associations between age, SOFA score, APACHE IV, BUN, Scr, FPG, TG and 28-day all-cause mortality, all significant at *p* < 0.05. Conversely, a negative association was observed with HDL-C and GCS score (all *p* < 0.05; detailed in Table 2).

**Table 2:** Factors influencing risk of ICU and hospital mortality analyzed by univariate Cox proportional hazards regression analysis.

Variable	Statistics	HR (95% CI)	*p*-value
Age (year)	63.61 ± 15.65	1.02 (1.01, 1.02)	<0.0001
BMI (kg/m^2^)	29.46 ± 8.01	1.00 (0.99, 1.00)	0.3541
Gender			
Male, *n* (%)	4,945 (42.66%)	1.0	
Female, *n* (%)	6,646 (57.34%)	0.94	0.3771
Ethnicity			
Caucasian, *n* (%)	8,287 (72.27%)	1.0	
African-American, *n* (%)	1,617 (14.10%)	0.70 (0.57, 0.86)	0.0009
Hispanic, *n* (%)	773 (6.74%)	0.74 (0.54, 1.02)	0.0641
Asian, *n* (%)	514 (4.48%)	0.80 (0.57, 1.11)	0.1771
Native American, *n* (%)	67 (0.58%)	0.63 (0.20, 1.97)	0.4278
Unknown, *n* (%)	209 (1.82%)	0.80 (0.46, 1.39)	0.4353
SOFA score, median (IQR)	1.00 (1.00–4.00)	1.32 (1.29, 1.35)	<0.0001
GCS score, median (IQR)	15.00 (130–15.0)	0.82 (0.81, 0.84)	<0.0001
APACH IV, median (IQR)	47.0 (35.0–63.0)	1.03 (1.03, 1.04)	<0.0001
BUN, median (IQR), (mmol/L)	17.0 (12.0–26.0)	1.01 (1.01, 1.01)	<0.0001
Scr, mg/dL	1.37 ± 1.53	1.07 (1.04, 1.10)	<0.0001
FPG, median (IQR), (mg/dL)	123.0 (102.0–165.0)	1.00 (1.00, 1.00)	<0.0001
TC, (mg/dL)	157.06 ± 52.72	0.99 (0.99, 1.00)	<0.0001
TG, median (IQR), (mg/dL)	108.0 (75.0–163.0)	1.00 (1.00, 1.00)	0.0291
HDL, (mg/dL)	42.52 ± 16.27	0.99 (0.99, 1.00)	0.0026
TyG index	8.96 ± 0.83	1.13 (1.04, 1.21)	0.0022
Medical history			
DM, *n* (%)	1,291 (11.14%)	0.96 (0.78, 1.19)	0.7391
COPD, *n* (%)	599 (5.17%)	0.90 (0.67, 1.20)	0.4673
CHF, *n* (%)	1,001 (8.63%)	1.18 (0.95, 1.45)	0.1344
AMI, *n* (%)	2,414 (20.82%)	0.84 (0.68, 1.02)	0.0826

CI, confidence interval; HR, hazard ratios.

### Association between TyG index and 28-day all-cause mortality

We utilized three Cox proportional hazards regression models to assess the impact of the TyG index on 28-day all-cause mortality in ED critically ill patients. In the initial unadjusted model (Model I), a 1-unit increase in the TyG index was associated with a 13% increase in the likelihood of all-cause mortality, with a HR of 1.13 (95% CI: 1.04–1.21, *p* = 0.0022). In the partially adjusted model (Model II), which only considered gender, age, race, and BMI, each 1-unit increase in the TyG index was linked to a 25% higher mortality risk, yielding an HR of 1.25 (95% CI: 1.15–1.35, *p* < 0.0001). The fully adjusted model (Model III) demonstrated that for each 1-unit increase in the TyG index, the risk of patient mortality increased by 19%, resulting in an HR of 1.19 (95% CI: 1.04–1.35, *p* = 0.0089). Consistent confidence intervals further support the strong relationship between the TyG index and 28-day mortality in emergency critically ill patients, as illustrated in Table 3.

**Table 3:** Association between TyG index and all-cause mortality in different models.

Exposure	Model I (HR, 95%CI) *p*	Model II (HR, 95%CI) *p*	Model III (HR, 95%CI) *p*
TyG index	1.13 (1.04, 1.21) 0.0022	1.25 (1.15, 1.35) < 0.0001	1.19 (1.04, 1.35) 0.0089
(TyG index Tertile)			
T1	Ref	Ref	Ref
T2	1.22 (1.02, 1.47) 0.0305	1.24 (1.03, 1.50) 0.0227	1.32 (1.04, 1.67) 0.0219
T3	1.56 (1.31, 1.86) < 0.0001	1.85 (1.54, 2.22) < 0.0001	1.56 (1.21, 2.02) 0.0007
*p* for trend	<0.0001	<0.0001	0.0007

Model I: we did not account for additional variables.

Model II: we adjusted gender, age, ethnicity and BMI.

Model III: we adjusted gender, age, ethnicity, BMI, SOFA score, GCS score, APACHE IV, BUN, Scr, TC, HDL, COPD, CHF, AMI, and DM. HR, Hazard Ratios; CI, confidence; Ref, reference.

Additionally, we transformed the TyG index into a categorical variable and reintroduced it into our model. The multivariate-adjusted model revealed that compared to participants in the first tertile (T1), the HR for those in the second and third tertiles (T2–T3) were 1.32 (1.04–1.67) and 1.56 (1.21–2.02), respectively. This indicates that participants in T2 and T3 had a 32 and 56% increased likelihood of all-cause mortality, respectively (see Table 3, Model III).

### Sensitivity analyses

Table 4 presents a sensitivity analysis of the association between the TyG index and all-cause mortality. In Model I, the sensitivity analysis focused on Caucasian patients (*N* = 8,287). After adjusting for confounding variables, the TyG index was associated with an increased risk of all-cause mortality, yielding a HR of 1.21 (95% CI: 1.04–1.40, *p* = 0.0109). Model II focused on patients aged 45 years and older (*N* = 10,322). In this model, the TyG index was significantly associated with an increased risk of all-cause mortality, with an HR of 1.25 (95% CI: 1.09–1.43, *p* = 0.0012). Model III excluded patients with a BMI greater than 25 kg/m^2^ (*N* = 7,855). In this model, the TyG index was associated with an HR of 1.20 (95% CI: 1.03–1.41) for all-cause mortality (*p* = 0.0225). These extensive sensitivity analyses emphasize the reliability of our findings (see Table 4). Additionally, we generated an E-value to assess sensitivity to unmeasured confounding. An E-value of 1.68 is required to explain away the observed HR of 1.19.

**Table 4:** Association between TyG index and all-cause mortality in different sensitivity analyses.

Exposure	Model I (HR, 95%CI) *p*	Model II (HR, 95%CI) *p*	Model III (HR, 95%CI) *p*
TyG index	1.21 (1.04, 1.40) 0.0109	1.25 (1.09, 1.43) 0.0012	1.20 (1.03, 1.41) 0.0225
(TyG index Tertile)			
T1	Ref	Ref	Ref
T2	1.37 (1.05, 1.79) 0.0223	1.40 (1.10, 1.79) 0.0065	1.15 (0.85, 1.55) 0.3763
T3	1.52 (1.13, 2.04) 0.0057	1.69 (1.29, 2.20) 0.0001	1.56 (1.13, 2.14) 0.0062
*p* for trend	0.0062	0.0001	0.0047

Model I was a sensitivity analysis performed with Caucasian patients (*N* = 8,287). We adjusted gender, age, BMI, SOFA score, GCS score, APACHE IV, BUN, Scr, TC, HDL, COPD, CHF, AMI, and DM.

Model II was a sensitivity analysis performed patients’ age ≥ 45 years (*N* = 10,322). We adjusted gender, age, ethnicity, BMI, SOFA score, GCS score, APACHE IV, BUN, Scr, TC, HDL, COPD, CHF, AMI, and DM. Model III was a sensitivity analysis performed patients’ BMI ≥ 25 kg/m^2^ (*N* = 7,855). We adjusted gender, age, ethnicity, BMI, SOFA score, GCS score, APACHE IV, BUN, Scr, TC, HDL, COPD, CHF, AMI, and DM.

### Identification of nonlinear association

**Figure 4:**
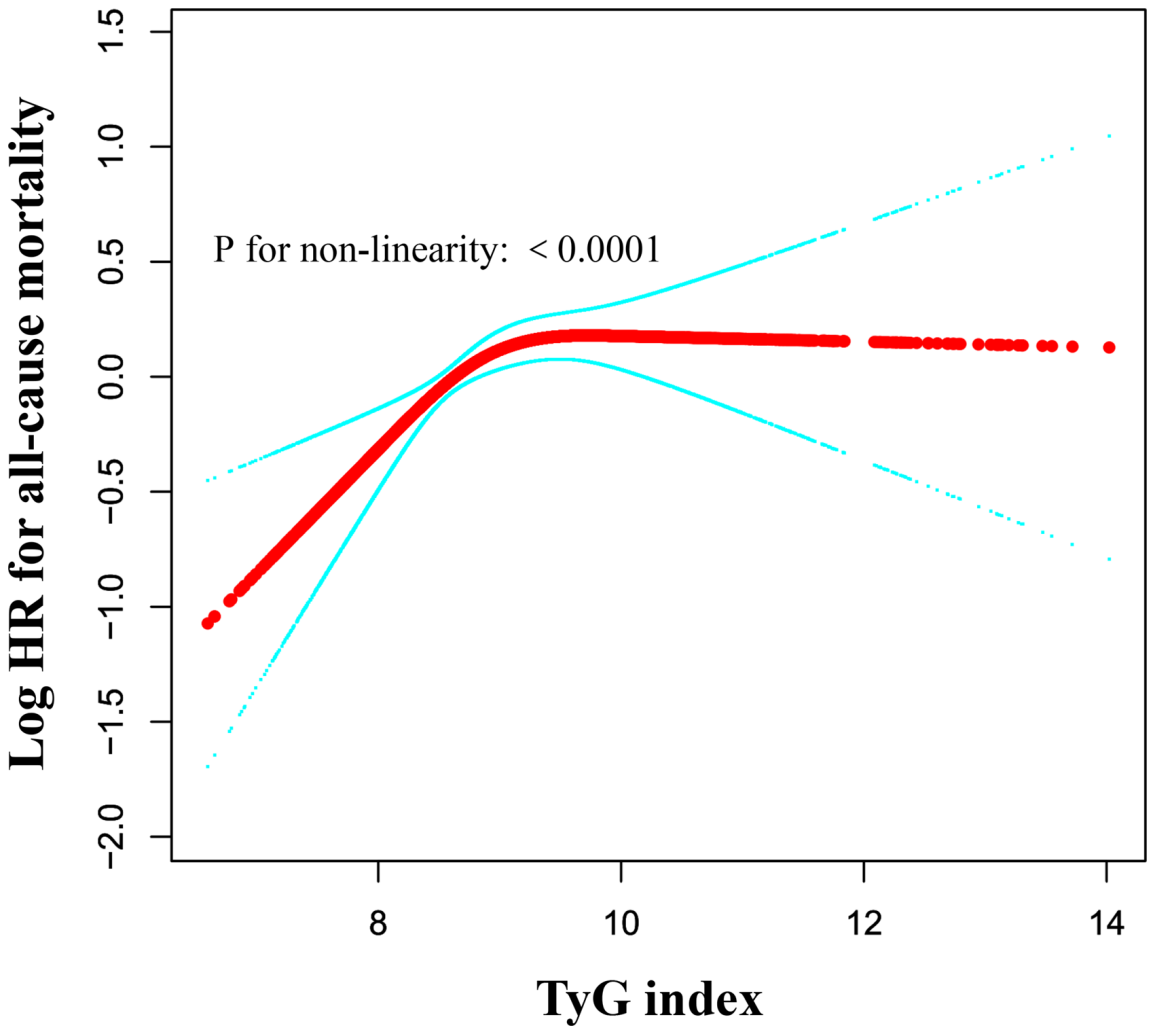
Associations between TyG index and all-cause mortality in critically ill patients. A threshold, nonlinear association between TyG index and all-cause mortality was identified using a generalized additive model (GAM). The solid red line represents the smooth curve fitted between the variables. The blue bands represent the 95% CI from the fitted model. The analysis was adjusted for gender, age, ethnicity, BMI, SOFA score, GCS score, APACHE IV, BUN, Scr, TC, HDL, COPD, CHF, AMI, and DM.

We utilized a generalized additive model and found a nonlinear saturation effect relationship between the TyG index and all-cause mortality (see Figure 4). We compared the linear regression model with a two-piecewise linear regression model, and the log-likelihood ratio test yielded a *p*-value of 0.001. This result indicates that the two-piecewise linear regression model should be used for model fitting (see Table 5). In other words, when the TyG index is less than 9.84, each 1-unit increase in the TyG index is associated with a 42% increase in all-cause mortality (adjusted HR 1.42, 95% CI 1.20–1.69, *p* < 0.0001). When the TyG index is ≥9.84, the mortality rate in critically ill patients no longer increases with increasing TyG index (adjusted HR 1.04, 95% CI 1.03–1.05, *p* = 0.0972).

**Table 5:** Threshold effect analysis of the TyG index and all-cause mortality.

Outcome: all-cause mortality	HR, 95%CI	*p*-value
Standard Cox regression	1.19 (1.04, 1.35)	0.0089
Two-piecewise Cox regression		
Inflection points of TyG index	9.84	
<9.84	1.42 (1.20, 1.69)	<0.0001
≥9.84	1.04 (1.03, 1.05)	0.0972
*p* for log-likelihood ratio test*	0.001	

Data were presented as HR (95% CI) p value; Model I, linear analysis; Model II, non-linear analysis. Adjusted for gender, age, ethnicity, BMI, SOFA score, GCS score, APACHE IV, BUN, Scr, TC, HDL, COPD, CHF, AMI, and DM. CI, confidence interval; OR, odds ratio; LRT, logarithm likelihood ratio test. *p < 0.05 indicates that model II is significantly different from Model I.

### Subgroup analyses

**Figure 5:**
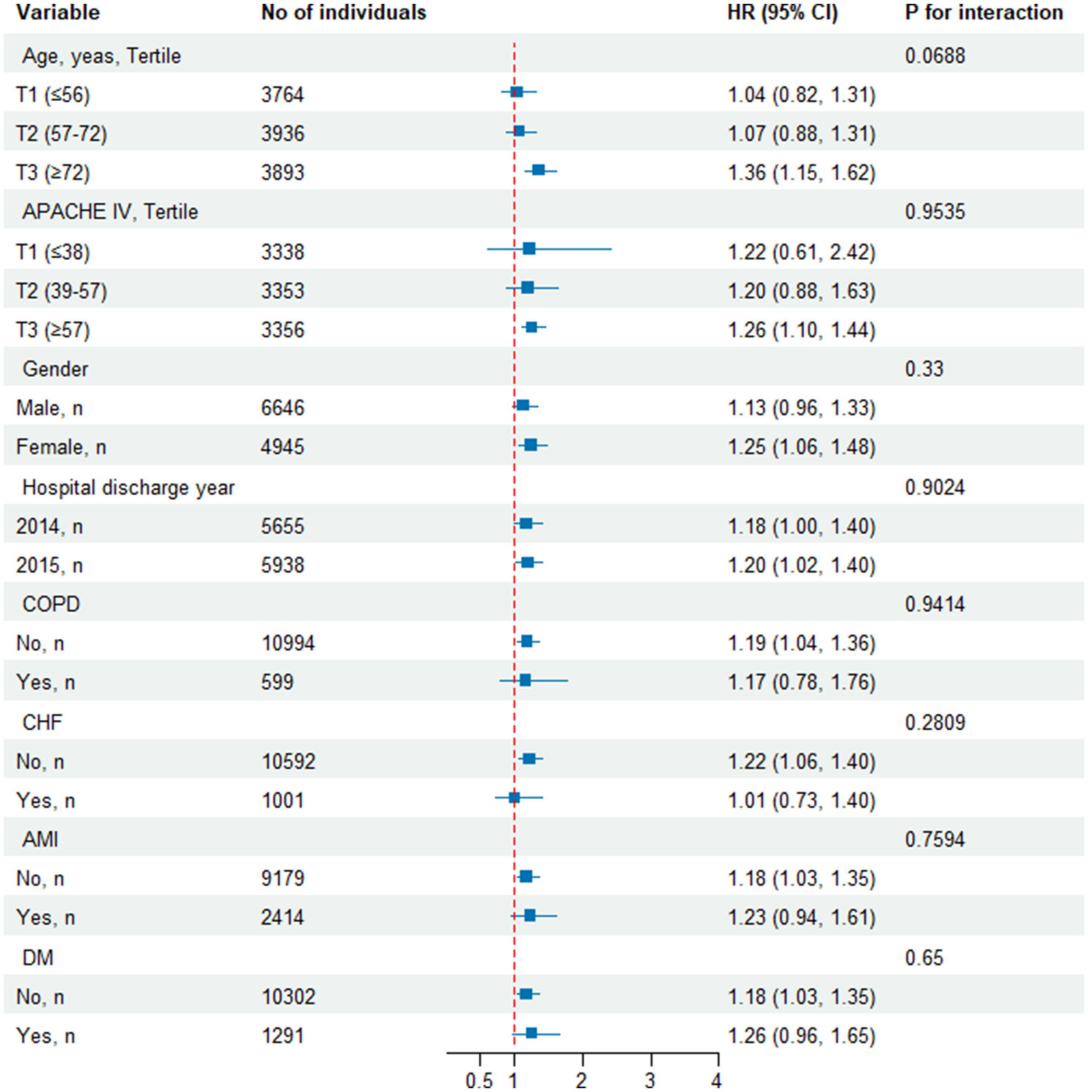
Effect size of TyG index on all-cause mortality in prespecified and exploratory subgroups. Above model adjusted for gender, age, ethnicity, BMI, SOFA score, GCS score, APACHE IV, BUN, Scr, TC, HDL, COPD, CHF, AMI, and DM. In each case, the model is not adjusted for the stratification variable when the stratification variable was a categorical variable.

As shown in Figure 5, we conducted subgroup analyses to evaluate the association between the TyG index and all-cause mortality. The primary findings indicated that in patients aged over 72 years, the TyG index was highly correlated with all-cause mortality. Additionally, a strong association between the TyG index and all-cause mortality was observed in female patients, and those without CHF. The study did not find significant interaction effects among the subgroups (all interaction *p*-values were greater than 0.05).

## Discussion

This multicenter study establishes the TyG index as a time-sensitive prognostic tool for 28-day mortality in ED-to-ICU patients. We identified a nonlinear threshold effect (inflection point: 9.84), where mortality risk escalates sharply below this cutoff (HR = 1.47) but plateaus above it—a critical insight for ED risk stratification. Unlike traditional ICU scores requiring delayed multi-organ assessments (e.g., APACHE IV), the TyG index leverages two routine ED biomarkers (FPG and TG), enabling risk calculation by short time after emergency admission (32, 33). This aligns with the “golden hour” principle in emergency care, where rapid triage directly impacts outcomes.

Given the simplicity, operability, and high efficiency of the TyG index in various clinical applications, it has been widely adopted in clinical practice. Numerous studies have shown that the TyG index is closely associated with various metabolic disorders, cardiovascular diseases, and the occurrence and prognosis of atherosclerosis (34–41). Many clinical studies have found a significant correlation between elevated TyG index levels and increased hospital mortality rates in critically ill patients, effectively predicting poor outcomes in cases of cardiac arrest, stroke, trauma, coronary artery disease, and sepsis. For instance, Cai et al. (24) extracted data from the MIMIC-IV database involving 733 patients with severe ischemic stroke and found a significant association between elevated TyG index and in-hospital and ICU all-cause mortality using Cox proportional hazards regression analysis. Similarly, Zheng et al. (18) explored the prognostic role of the TyG index for the first time in critically ill infectious patients using a retrospective observational cohort study involving 1,257 severe infection patients from the MIMIC-IV database, indicating a correlation between elevated TyG index and increased in-hospital mortality in these patients. Ye et al. (42) investigated 639 patients with chronic kidney disease and coronary artery disease in the ICU, discovering that the TyG index could predict in-hospital and one-year mortality rates. Also, Zhang et al. (43) analyzed 1,618 severe coronary artery disease patients from the MIMIC-III database, highlighting that the TyG index could significantly predict hospital and ICU mortality rates in these patients. Huang et al. (44) indicated that the TyG index can serve as a novel assessment tool for predicting all-cause mortality in critically ill hemorrhagic stroke patients, noting that it can predict mortality due to hemorrhagic stroke. However, the patients included in these studies may have come from various departments and not solely from the emergency department, and several limitations exist within these studies: (i) Some studies utilized retrospective designs, which may lead to selection bias and information bias; (ii) Smaller sample sizes (such as 733 patients) may limit the generalizability of the results; (iii) Despite considering several confounding variables, there may still be unrecognized or uncontrolled confounding factors that could bias the results.

To address the deficiencies and limitations mentioned above, we conducted a multicenter retrospective cohort study based on the eICU database, including 11,593 ED-to-ICU critically ill patients, and investigated the association between the TyG index and the 28-day mortality rate of ED-to-ICU critically ill patients. Moreover, we generated an E-value to assess the sensitivity to unmeasured confounding factors. Our results indicated that the high TyG index group had significantly higher 28-day in-hospital all-cause mortality compared to the low TyG index group. The TyG index exhibited a significant positive correlation with 28-day all-cause mortality rates, and this association remained significant after adjusting for various clinical and laboratory variables. Furthermore, we utilized a cubic spline function in the Cox proportional hazards regression model to identify a nonlinear saturation effect, with an inflection point at 9.84. This finding indicates that an increased TyG index significantly elevates the risk of mortality, particularly when exceeding this threshold. Below this inflection point, all-cause mortality increases with rising TyG index levels; above this point, all-cause mortality reaches a saturation level (see Table 5).

Furthermore, our study conducted risk stratification analysis across various subgroups. The subgroup analysis indicated that in patients aged over 72 years, the TyG index was a more significant predictor of 28-day all-cause mortality, consistent with previous studies (45). This highlights the need to pay closer attention to older patients, as they are likely to experience more complications and a faster progression of illness. In females, the TyG index seemingly predicts 28-day all-cause mortality more significantly, potentially related to higher age, SOFA score, and APACHE IV, along with a greater prevalence of COPD and CHF in this cohort compared to males (Supplementary Table 1). Another interesting finding in our study was that among Caucasians, the TyG index was also a very significant predictor of 28-day all-cause mortality, which may be related to their metabolic characteristics, dietary habits, and responses to cardiovascular risk factors. However, we observed a connection between the TyG index and all-cause mortality in patients without CHF. This phenomenon may be attributed to reverse causation: patients diagnosed with these comorbidities are more likely to receive appropriate treatment and greater attention. Therefore, although their overall mortality risk is higher, their prognosis may be improved. Furthermore, in our subgroup analysis, we did not observe any significant interactions. This indicates that the relationship between the TyG index and all-cause mortality is consistent across different subgroups and is not significantly influenced by other variables. This finding enhances our confidence in the applicability of the TyG index in clinical settings and suggests its potential as a risk assessment tool.

Evidently, our primary results align similarly with previous studies. Huiruo Liu et al. (46) investigated the association between the TyG index and outcomes in patients with acute myocardial infarction complicated by cardiogenic shock (AMICS), including 375 patients who developed cardiogenic shock. The study found a significant elevation of the TyG index in these patients, with a linear correlation to both in-hospital and ICU mortality rates. Furthermore, this study indicated a cutoff value of 9.22, above which patients displayed a significantly increased risk of death during hospitalization and in the ICU. Additionally, Ying Liao et al. (47) analyzed 3,026 critically ill patients and exhibited a non-linear association between the TyG index and mortality, with a cutoff point of 9.2. However, compared to our study, these earlier investigations often did not adequately consider the specificity of the TyG index and its potential application in emergency critically ill patients. Our study identified a non-linear association between the TyG index and all-cause mortality among ED-to-ICU critically ill patients, with a cutoff point of 9.84, which is slightly higher than previous findings. This indicates that the TyG index for all-cause mortality to reach saturation is higher in ED critically ill patients. This phenomenon may be attributed to the presence of multiple comorbidities and the complex clinical conditions of these patients. Furthermore, their studies had relatively small sample sizes and lacked multicenter data support, which may limit the validity of the results. In other words, the findings of this study are more reliable and worthy of widespread clinical application. On the other hand, some studies may have focused on other biomarkers or scoring systems, such as APACHE II or SOFA scores (48). These scoring systems typically require multiple data inputs, which may be challenging to obtain swiftly within an emergency setting, delaying patient risk assessment. In contrast, the TyG index is relatively simple to compute and can be rapidly acquired in the emergency department, making it suitable for immediate applications in clinical practice.

Although the mechanisms underlying the close association between the TyG index and all-cause mortality in ICU patients are not yet fully elucidated, the prevailing hypothesis suggests that metabolic abnormalities due to IR could be a significant contributor (47). Firstly, IR can lead to metabolic disorders, such as hyperglycemia and abnormal lipid metabolism, potentially exacerbating the underlying conditions of critically ill patients. These metabolic abnormalities are closely linked to an increased risk of complications, including infections, inflammation, and tissue hypoperfusion, which can further worsen the condition (49, 50). Secondly, the TyG index has been shown to amplify inflammatory responses, oxidative stress, endothelial dysfunction, and cardiovascular remodeling, all of which are primary contributors to the deterioration of critically ill patients’ health (51, 52). Through these mechanisms, a close association between the TyG index and overall mortality rates in critically ill patients is observed.

Our study possesses several strengths: Firstly, it is the first to verify a significant positive correlation between the TyG index and increased 28-day all-cause mortality rates in ED-to-ICU critically ill patients. Additionally, it identifies a nonlinear saturation effect association between the TyG index and 28-day all-cause mortality. Secondly, this study utilizes a large sample size of 11,593 patients and employs a multicenter retrospective cohort design, contrasting with many previous studies characterized by small sample sizes or case–control designs, thus enhancing the representativeness and broad applicability of the findings. Thirdly, our analysis considers various potential confounding factors (e.g., gender, age, BMI, SOFA score, GCS score and APACH IV, etc.), utilizing E-values to assess sensitivity to unmeasured confounders, revealing robust results. Many earlier studies failed to adequately adjust these factors, potentially leading to bias in their results. Fourthly, our study conducted thorough sensitivity analyses to validate the robustness of the TyG index as a predictor of mortality risk, bolstering the credibility of the findings. Fifthly, it included subgroup analysis and interaction evaluations. Through subgroup analysis, the study identified associations between the TyG index and all-cause mortality rates across different ages, APACHE IV, genders, or underlying disease states (e.g., DM, CHF, COPD, and AMI). This analysis aids in determining which specific populations might be more affected by the TyG index, thus providing more targeted guidance for clinical decision-making. Interaction analysis across various subgroups further validated whether the predictive capability of the TyG index remained consistent across diverse populations. This method enhances the robustness of the findings, making the study conclusions more widely applicable.

However, several limitations and shortcomings must be acknowledged in this research. Firstly, as a retrospective study, it may be subject to selection bias and information bias, which could affect the interpretation of the results. Secondly, although the study controlled for various confounding factors, there may still be unrecognized confounding variables. We utilized the E-value sensitivity analysis to quantify the potential implications of unmeasured confounders and found that an unmeasured confounder was unlikely to fully explain the treatment effect. Thirdly, this study experienced some missing data; although multiple imputation methods were employed to address this issue, it may still influence the accuracy of the results. Fourthly, the study did not delve deeply into the biological mechanisms linking the TyG index and mortality rates in ED-to-ICU critically ill patients, suggesting an area for future research to explore potential physiological mechanisms further. Fifthly, due to database constraints, we could not confirm whether all glucose measurements were derived from fasting samples. Sixthly, while the findings of this study demonstrate a degree of representativeness, further validation in other independent cohorts is necessary to confirm their generalizability. Seventhly, this study is based on the eICU database, which mainly covers data from hospitals in the United States. Therefore, we suggest that caution should be exercised when applying the TyG index threshold derived in this study to populations outside the United States. Eighthly, our analysis considers various potential confounding factors (e.g., gender, age, BMI, SOFA score, GCS score and APACHE IV, etc.), utilizing E-values to assess sensitivity to unmeasured confounders. However, this alone is not sufficient. We will consider further detailed exploration of specific potential confounders, such as the use of vasopressors and fluid resuscitation, and how they might influence the association between the TyG index and mortality. This will help to provide a more comprehensive understanding of the limitations and robustness of our findings. Finally, the data used in this study originated from the United States, which means the results may not be fully applicable to ICUs in other countries. Despite potential limitations arising from regional differences, the characteristics revealed by this study can serve as a reference for patient management and treatment planning in other regions, contributing to the improvement of critical care quality on a global scale.

## Conclusion

In ED-to-ICU critically ill patients, the TyG index demonstrates a nonlinear relationship with 28-day mortality, serving as a rapid, actionable biomarker for emergency risk stratification. A threshold of 9.84 identifies high-risk patients requiring immediate metabolic optimization (e.g., glycemic control, lipid management) and prioritized ICU admission.

## Data Availability

The datasets presented in this study can be found in online repositories. The names of the repository/repositories and accession number(s) can be found in the article/Supplementary material.

## Footnotes

^1^ www.empowerstats.com

^2^ http://www.r-project.org
