# Supplementary material for: Emergency risk stratification using the TyG index: a multi-center cohort study on nonlinear association with 28-day mortality among critically ill patients transferred from the ED to the ICU
Source: Front Med (Lausanne). 2025 Jul 30;12:1605843. doi: 10.3389/fmed.2025.1605843 (PMC12343646; doi:10.3389/fmed.2025.1605843)
Supplement: Supplementary file 1 [file Table_1.docx]

Association Between Triglyceride-glucose Index and All-Cause Mortality in Critically Ill Patients from Emergency Department: A Multi-Center Cohort Study

Supplement **Table 1: Differences in Age, SOFA Score, COPD, CHF, and WBC Between Genders**

| **Variable** | Female | Male | P value |
| --- | --- | --- | --- |
| Age, years | 65.97 ± 16.08 | 61.85 ± 15.08 | <0.001 |
| N | 4,945 | 6,646 |  |
| COPD, n (%) | 292 (5.90%) | 307 (4.62%) | 0.002 |
| CHF, n (%) | 483 (9.77%) | 518 (7.79%) | <0.001 |
| SOFA score, median (IQR) | 2.00 (1.00-4.00) | 1.00 (0.00-4.00) | 0.009 |
| APACH IV, median (IQR) | 49.0 (38.0-65.0) | 44.0 (33.0-61.0) | <0.001 |
